# Oxidative stability of canola oil by *Biarum bovei* bioactive components during storage at ambient temperature

**DOI:** 10.1002/fsn3.560

**Published:** 2017-12-06

**Authors:** Reza Farahmandfar, Mohammad Hossein Ramezanizadeh

**Affiliations:** ^1^ Department of Food Science and Technology Sari Agricultural Sciences & Natural Resources University Sari Iran; ^2^ Department of Food Science and Technology Sari Branch Islamic Azad University Sari Iran

**Keywords:** Antioxidant activity, *Biarum bovei*, canola oil, oxidative stability, phenolic compounds

## Abstract

In this study, antioxidative activities of aqueous extract of *Biarum bovei* (BBE) in stabilizing of canola oil during storage at 20°C was evaluated. For this purpose, the total phenolic (TP), flavonoids (TFC), and tocopherol content (TTC) of the extract were determined and β‐carotene bleaching system was used to assess the antioxidant efficacy of BBE. The amount of TP, TFC, and TTC in BBE indicated high antioxidant activity. So, different concentrations (0, 200, 800, and 1400 ppm) of BBE and butylatedhydroxyanisole (BHA; 100 ppm), were added to canola oil for 60 days at 20°C. Peroxide value (PV), carbonyl value (CV), Total polar compounds (TPC), acid value (AV), iodine values (IV), and conjugated dienes (CD) were employed to evaluate the BBE effect on canola oil stabilizing. Results indicated that 1,400 ppm of BBE exhibited stronger antioxidant activity in canola oil than BHA.

## 1 INTRODUCTION

Canola oil is one of the best nutritious edible oils in the whole world, due to its high content of polyunsaturated fatty acids (PUFA) and it is a rich resource of vitamin E (Farahmandfar, Asnaashari, & Sayyad, 2015). Lipid oxidation is the major cause of oil quality deteriorating in food industry. The rate of oxidation of fats and oils is affected by the light and heat, oxygen partial pressure, trace metal, and the degree of unsaturation of fatty acids (Asnaashari, Farhoosh, & Sharif, 2014). Thus, the use of antioxidants to retard or prevent the oxidative deterioration of oils in food product is extensively practiced.

The addition of synthetic antioxidants such as butylatedhydroxytoluene (BHT), butylatedhydroxyanisole (BHA), ter‐butyl hydroquinone (TBHQ) have been used to slow down the oxidation process over 50 years (Eshghi, Asnaashari, Haddad Khodaparast, & Hosseini, 2014). However, the use of synthetic antioxidants has been limited by reason of being toxic and carcinogenic to humans. The most powerful synthetic antioxidant (TBHQ) is forbidden to use in Europe, Japan, and Canada. Moreover, BHT has also been removed from the generally recognized as safe (GRAS) list of compounds (Asnaashari, Tajik, & Khodaparast, 2015; Asnaashari et al., 2014). Nowadays, there has been increased interest in identifying the potential sources of natural antioxidants (Farahmandfar et al., 2015; Poiana, 2012). Therefore, plant extracts which provide bioactive components have been reported to be more effective than some major synthetic antioxidants. Extracts from spices, herbs, fruits, and vegetables contain effective bioactive compounds preventing from oxidative deterioration in food product (Farahmandfar et al., 2015).

*Biarum bovei* belongs to Araceae family, *Biarum* genus. The genus *Biarum* comprises 21 species of dwarf tuberous‐stemmed herbs distributed from Portugal to Iran (Boyce, 1987). The presence of flavonoids, alkaloids, anthocyanins, amines, and cinnamic acids in Araceae family have been reported by some researchers (Hegnauer, 1987; Williams, Harborne, & Mayo, 1981). These compounds, due to having antioxidant activity, are capable of scavenging free radicals. Consequently, they could be effective in preventing oxidative off‐flavor and deterioration of oils in food industry.

There is no report describing efficiency of *B. bovei* extract for the stabilization of food materials, especially canola oil. The purpose of this study was to determine the effect of *B. bovei* extract on the stabilization of canola oil and comparing its antioxidant activity with commercially available antioxidant (BHA) during storage at 20 ± 1°C.

## 2 MATERIALS AND METHODS

### 2.1 Materials

Refined‐bleached‐deodorized canola oil (without any synthetic antioxidants) was obtained from Ghoncheh Co. (Sari, Iran). The plant *B. bovei* was collected from the south of Iran (Izeh, Kozestan, Iran) in April 2016 and authenticated by a botanist (School of Pharmacy, Sari University of Medical Sciences, Sari, Iran). They were washed, air‐dried (in the shade) and powdered fine using an electric device then stored at 4 ± 1°C until use. All other chemicals and reagents were of analytical grade and purchased from Sigma‐Aldrich (St. Louis, MO, USA) and Merck (Frankfurt, Germany). BHA was also purchased from Sigma‐Aldrich (St. Louis, MO, USA).

### 2.2 Preparations of *B. bovei* extract (BBE)

The powdered *B. bovei* was added into water (1:50 wt/vol) and the resulting mixture was shaken in a dark place at room temperature for 24 hr. Then, the extract was filtered using Whatman No. 42 filter paper and residue was again extracted. The solvent was completely evaporated at 40°C. The obtained extract was weighted to calculate the yield and was stored in a dark container in refrigerator (4 ± 1°C) until used (Asnaashari et al., 2014).

### 2.3 Determination of total phenolic, flavonoids, and tocopherol content

Total phenolic content (TP) of extract was measured according to the Folin–Ciocalteu method and result was expressed as mg/g Gallic acid equivalents (GAE) (Farahmandfar et al., 2015). The total flavonoid content (TFC) of extract was determined according to the method described by Sayyari and Farahmandfar (2017) and the results were expressed as catechin equivalents (mg/100 g of dry weight). The total tocopherol content (TTC) was measured spectrophotometrically in keeping with method described by Asnaashari et al. (2014) and the results are expressed as microgram of α‐tocopherol equivalents in milliliter extract (μg α‐tocopherol/ml extract).

### 2.4 Antioxidant activity (β‐carotene bleaching system)

Lipid peroxidation inhibition activity of different concentrations of BBE was determined using the β‐carotene bleaching method (Farahmandfar et al., 2015). For this purpose, 0.5 mg β‐carotene was dissolved in 1 ml chloroform (high‐performance liquid chromatography grade). Then, 25 μl linoleic acid and 200 mg Tween 40 were added. Chloroform was completely evaporated using rotary vacuum evaporator. At that moment, 100 ml of distilled water saturated with oxygen was added and the contents were shaken vigorously, 2.5 ml of the above solution was transferred to the test tube and 350 μl of each extract (with a concentration of 2 g/L dissolved in their own solvent) was added. All samples were put into a water bath for 120 min at 50°C. The absorbance values of samples were read spectrophotometrically at 470 nm at zero time and after 120 min. Antioxidant capacity of the extracts were expressed by applying following equation:

(1) $$\text{Inhibition}\;\left(\%\right)\;=\;\frac{A_{\mathrm{control}}\;-\;A_{\mathrm{extract}}}{A_{\mathrm{control}}}\;\times \;100$$

where *A*_control_ is the absorbance of the control (without extract) and *A*_extract_ is the absorbance of extract.

### 2.5 Preparation of canola oil

The solubility of the water extract of a plant is low in oil. For that reason, we added 0.3% polyglycerol polyricinoleate (PGPR) content to the oily phase. To ensure complete solution, the oily phase with the respective amount of PGPR was stirred for 30 min with a magnetic stirrer at 300 rpm. While stirring, the aqueous phase was slowly added to prepare uniform solution. Therefore, canola oil containing different concentrations of BBE (0, 200, 800, and 1400 ppm) and 100 ppm of BHA as control antioxidant were placed in dark brown colored bottles and stored at 20 ± 1°C for 60 days. Samples (20 g) were removed periodically at specific interval (0, 15, 30, 45, and 60 days) for analysis.

### 2.6 Analytical procedures

Peroxide value (PV) was determined spectrophotometric as said by the method of Farhoosh, Johnny, Asnaashari, Molaahmadibahraseman, and Sharif (2016) and the results are expressed as meq O_2_/Kg oil.

The carbonyl value (CV) of the canola oil was assessed according to the method described by Farahmandfar et al. (2015) using 2‐propanol and 2, 4‐decadienal as solvent and standard, respectively, and the results are expressed as μmol/gr.

Acid value (AV) was determined in keeping with the method of Sayyari and Farahmandfar (2017) and the results are expressed as mg/g.

Iodine value (IV) was determined according to the method described by Eshghi et al. (2014) and the results are expressed as g Iodine/100 g oil.

Total polar compounds (TPC) content of canola oil was measured in line with the economical micro method described by Farahmandfar et al. (2015) and the results are expressed as %.

Conjugated diene value (CDV) was specified based on the method of Sharayei and Farhoosh (2016). Canola oil samples were diluted to 1:600 with hexane and measured spectrophotometrically at 234 nm. An extinction coefficient of 29,000 mol/L was used to quantify the concentration of conjugated dienes formed in canola oil samples during oxidation.

### 2.7 Statistical analysis

Analysis of variance (ANOVA) was used and means comparison was performed by Duncans’ new multiple range test. SPSS statistic program (SPSS 16.0 for Windows, SPSS Inc., Chicago, IL, USA) was used for data analysis. All analyses were performed in triplicate and data reported as means ± standard deviation (*SD*).

## 3 RESULTS AND DISCUSSION

### 3.1 Evaluation of bioactive compounds of *Biarum bovei*

The yield of *B. bovei* water extract was 25.16%. Hosseini, Rousta, Tabib Loghmany, and Mahmoudpour (2014) reported that yield of hydromethanolic extract of *Biarum carduchrum* was 17.47%. Phenolics, flavonoids, and tocopherols are the main considerable groups of bioactive components in plants and herbs that act as reducing agents, hydrogen donors. Therefore, they inhibit oxidation process in various biological molecules (Farahmandfar et al., 2015). Moreover, these components are responsible for great free radical scavenging and antioxidant activities of fruits and vegetables (Maleki, Ariaii, & Fallah, 2015). TP, TFC, and TTC of *B. bovei* extracts are presented in Table 1. The results show that amount of TP, TFC, and TTC in BBEs were 37.42 ± 1.33 mg GAE /g, 12.5 ± 1.4 mg/100 g, and 110.25 ± 1.88 μg α‐tocopherol/ml, respectively. The amount of TP of *B. bovei* extracts were higher than *B. carduchrum* reported by Hosseini et al. (2014). Hence, these results suggested that *B. bovei* could be used as valuable sources of phenolic, flavonoid, and tocopherol compounds in the food products, pharmaceutical, medical, and chemical industries. Several studies demonstrated that these compounds in the extract contribute to the antioxidant activity (Ignat, Volf, & Popa, 2011).

**Table 1:** Yield, total phenolic, flavonoids, and tocopherol content of *Biarum bovei* extract (BBE)

	Yield (%)	Total phenolic content (mg GAE/g)	Total flavonoid contents (mg/100 g)	Total tocopherol content (μg α‐tocopherol/ml)
BBE	25.16 ± 1.28	37.42 ± 1.33	12.5 ± 1.4	110.25 ± 1.88

### 3.2 Antioxidant activity by β‐carotene bleaching system

In this analyze, the oxidation of linoleic acid produces peroxyl free radicals by reason of the abstraction of hydrogen atom from diallylic methylene groups of linoleic acid. These free radicals will oxidize unsaturated β‐carotene. In the presence of an antioxidant such as bioactive components of plant extract and essential oil, β‐carotene could scavenge the free radicals formed in the system and retains its original yellow color (Farahmandfar et al., 2015). The inhibition percentage of antioxidant activity (β‐carotene bleaching system) of water extract of *B. bovei* is presented in Table 2. According to the results, different concentrations of BBE had antioxidant activity; this indicates that the presence BBE may hinder the extent of β‐carotene bleaching by scavenging free radicals (Farahmandfar et al., 2015; Sowndhararajan, Joseph, & Manian, 2013). Furthermore, the antioxidant activity increased with increasing amount of BBE concentration. So, 1,400 ppm of *B. bovei* water extract exhibited the maximum antioxidant activity.

**Table 2:** Antioxidant activity (β*‐*carotene bleaching system) of different concentration of *Biarum bovei* extracts (BBE)

	% Inhibition					
	200 ppm	500 ppm	800 ppm	1,100 ppm	1,400 ppm	1,700 ppm
BBE	20.68 ± 1.55^f^	30.61 ± 2.86^e^	40.02 ± 2.65^d^	52.25 ± 1.05^c^	71.98 ± 2.13^a^	58.43 ± 4.23^b^

Means within a row with the different lowercase letters are significantly different at *p* < .05.

### 3.3 Oxidative stability of canola oil samples as affected by addition of BBE

**Figure 1:**
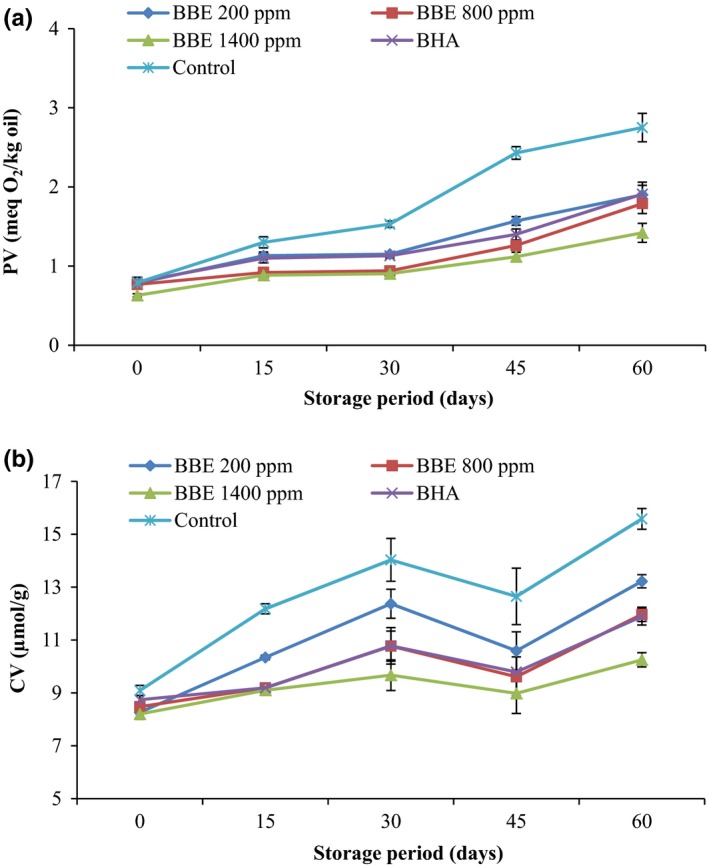
Effect of *Biarum bovei* extract (BBE) and BHA on peroxide value (PV) (a) and carbonyl value (CV) (b) of canola oil during storage at ambient temperature (Error bars show the variations of three determinations in terms of standard deviation) (♦, BBE 200 ppm; ■, BBE 800 ppm; ▲, BBE 1,400 ppm; ×, BHA; 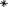, Control)

PV is an index for peroxides and hydroperoxides measurement produced in the initial stages of lipid oxidation. Besides, it is one of the most widely‐used methods for oils oxidative rancidity evaluation (Farhoosh et al., 2016). Figure 1a shows the PV changes of the canola oil samples including BHA and different concentrations of BBE during storage process at 20°C. A continuous increase in PV with the increase in storage time was observed for all the samples. Similar results have been reported by researchers (Eshghi et al., 2014; Sayyari & Farahmandfar, 2017).

Control samples (canola oil without the antioxidant) reached a maximum PV (2.75 meq O_2_/Kg oil) after 60 days of storage period. A significant difference (*p* < .05) in PV was observed between the control and canola oil samples containing different concentrations of BBE and BHA, which slowed the rate of peroxides and hydroperoxides formation revealing good antioxidant efficacy in stabilizing of canola oil. Moreover, as the concentration of the BBE increased, the oxidation inhibition of the extract also enhanced. At the end of storage period, the lowest amount of PV was observed in the canola oil containing 1,400 ppm *Biarum bovei* extract. The results demonstrated that *Biarum bovei* extract (at 1,400 ppm) had higher antioxidant activity than BHA. The results are in agreement with the results of other researchers (Asnaashari et al., 2015; Zhang et al., 2010).

CV is a good index of oxidative rancidity in oils and fats that estimates the content of secondary products of oxidation products such as aldehydes and ketones. Peroxides and hydroperoxides are transformed into secondary products, which are more stable than peroxides (Asnaashari, Farhoosh, & Farahmandfar, 2016). Figure 1b shows the CV changes of the canola oil samples including BHA and different concentrations of BBE during storage process at 20°C. There was a regular pattern of increase in CV for all the canola oil samples at most of the storage period, CV of canola oil samples increased until 30 days of storage and after that the content of CV reduced; the decrease in the CV content (after 45 days) was attributed to the decomposition of carbonyl compounds during the prolonged storage period and production of new components that were not detectable by the CV assay (Farhoosh & Kenari, 2009). A similar trend same as PV was also observed at the end of the storage period. So, the highest and lowest amount of CV was in the control samples and canola oil sample containing 1,400 ppm BEE (*p*<0.05). As it is mentioned above, the minimum amount of CV in the canola oil samples comprising BEE demonstrated a good antioxidant efficacy of extract. Similar results have been reported by researchers (Farahmandfar et al., 2015; Sayyari & Farahmandfar, 2017).

**Figure 2:**
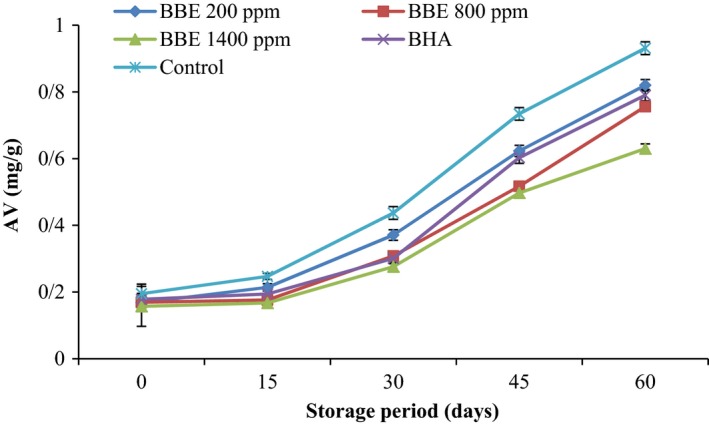
Effect of *Biarum bovei* extract (BBE) and BHA on acid value (AV) of canola oil during storage at ambient temperature (♦, BBE 200 ppm; ■, BBE 800 ppm; ▲, BBE 1,400 ppm; ×, BHA; 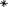, Control)

AV of the canola oil samples as affected by BHA and different concentrations of BBE during storage process at 20°C are shown in Figure 2. A continuous rise in AV with increase in storage period was observed for all the samples. The steady increase in the AV can be attributed to hydrolysis of triacylglycerols and to the component carboxyl groups present in oxidation process (Farahmandfar et al., 2015; Maleki et al., 2015; Nasirullah, 2001). During storage period, the highest amount of AV was observed in the control samples and the lowest amount of AV was observed in the canola oil sample containing BEE at 1,400 ppm, which suggests the superiority of the BEE over the synthetic antioxidants in delaying oxidation in canola oil.

**Figure 3:**
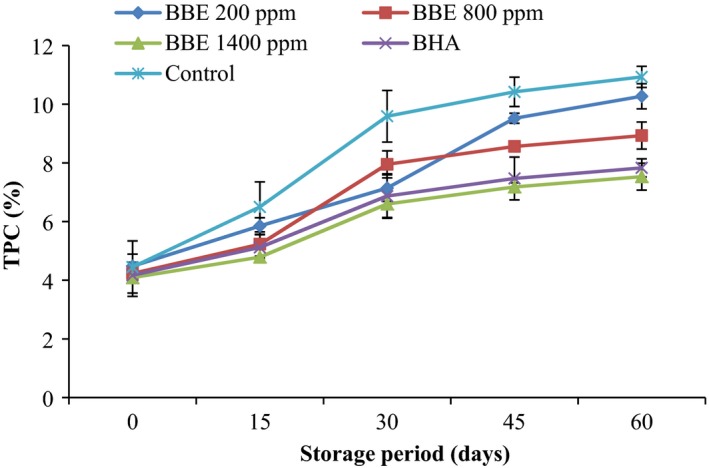
Effect of *Biarum bovei* extract (BBE) and BHA on total polar compounds (TPC) of canola oil during storage at ambient temperature (♦, BBE 200 ppm; ■, BBE 800 ppm; ▲, BBE 1,400 ppm; ×, BHA; 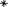, Control)

TPC is considered to be the most predominant indicator for the oil stability and quality evaluation. At all samples, as the storage time was increased, the TPC was also enhanced (Figure 3). At the end of storage time, TPC of control sample and canola oil samples including 200 ppm, 800 ppm, and 1,400 ppm BEE and samples including BHA was 10.93, 10.27, 8.93, 7.53, and 7.83, respectively. According to the results, the lowest amount of TPC was observed in canola oil contain 1,400 ppm BBE and BHA, there was no statistically significant difference between them (*p *> .05). Consequently, these samples could remarkably inhibit lipid oxidation in canola oil.

**Figure 4:**
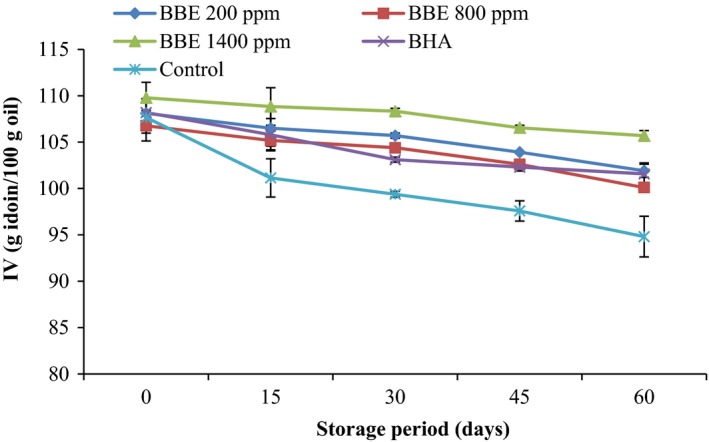
Effect of *Biarum bovei* extract (BBE) and BHA on iodine value (IV) of canola oil during storage at ambient temperature (♦, BBE 200 ppm; ■, BBE 800 ppm; ▲, BBE 1,400 ppm; ×, BHA; 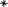, Control)

The IV of the canola oil samples containing antioxidants and the control samples are shown in Figure 4. A decreasing trend in the IV observed with increasing storage time for all the canola oil samples. As the storage time increases, the double bonds of the polyunsaturated fatty acids of canola oil are attacked by free radicals, and some of the double bonds were destroyed during autoxidation which results in reduction in IV. According to the Figure 4, the lowest amount of IV was observed in the control samples and the highest amount of IV was observed in the canola oil sample containing 1,400 ppm BEE (*p *< .05). Subsequently, it showed that the higher efficacy of BBE protect the polyunsaturated bonds of fatty acids in canola oil.

**Figure 5:**
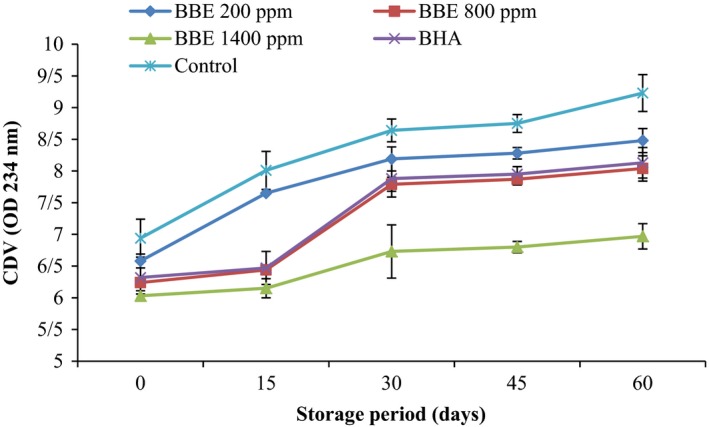
Effect of *Biarum bovei* extract (BBE) and BHA on conjugated diene value (CDV) of canola oil during storage at ambient temperature (♦, BBE 200 ppm; ■, BBE 800 ppm; ▲, BBE 1,400 ppm; ×, BHA; 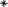, Control)

CDV is a good parameter for the oxidative deterioration measurement of oils, hence indicates the effectiveness of antioxidants in food products. As seen in Figure 5, the CDV raised with increasing storage period owing to formation hydroperoxides that possess conjugated diene structures. Increase in CDV in the presence of antioxidant is slightly lower than control samples. So that, at the end of storage period, the CDV of the control samples reached 9.23, whereas CDV of canola oil samples including 200, 800, and 1,400 ppm BEE and samples including BHA was 8.48, 8.04, 6.97, and 8.13, respectively. This demonstrated the antioxidant potential of BHA and BBE in stabilizing of canola oil. On the other hand, BHA with the higher CDV showed incompatible antioxidant properties in preventing lipid oxidation compared to BBE at 800 and 1,400 ppm.

## 4 CONCLUSION

In this study, it is found that *B. bovei* water extract had high TP, TFC, and TTC. Therefore, the antioxidant effect of this extract was evaluated to stabilize canola oil during storage at 20°C. The results could be summarized that all the concentrations of *B. bovei* extract can stabilize canola oil effectively. The antioxidant activity of *B. bovei* extract at concentration of 1,400 ppm generally greater than synthetic antioxidant (BHA) at its legal limit. Therefore, *B. bovei* water extract may be considered a source of natural antioxidant; hence, it may act as an alternative to synthetic ones for the stabilization of food formulation, possibly contributing to improve edible oils quality.

## CONFLICT OF INTEREST

The authors declare that there are no conflicts of interest.
